# Knowledge, attitude and practice towards family planning among reproductive age women in a resource limited settings of Northwest Ethiopia

**DOI:** 10.1186/s13104-018-3689-7

**Published:** 2018-08-13

**Authors:** Ayele Semachew Kasa, Mulu Tarekegn, Nebyat Embiale

**Affiliations:** 1 Department of Nursing, College of Medicine & Health Sciences, Bahir Dar University, Bahir Dar, Ethiopia; 2 Department of Surgery, School of Medicine, College of Medicine & Health Sciences, Bahir Dar University, Bahir Dar, Ethiopia

**Keywords:** Family planning, Knowledge, Attitude, Practice, Ethiopia

## Abstract

**Objective:**

To assess the knowledge and attitude regarding family planning and the practice of family planning among the women of reproductive age group in South Achefer District, Northwest Ethiopia, 2017.

**Result:**

The study showed that the overall proper knowledge, attitude and practice of women towards family planning (FP) was 42.3%, 58.8%, and 50.4% respectively. Factors associated with the practice of FP were: residence, marital status, educational status, age, occupation, and knowledge, and attitude, number of children and monthly average household income of participants. In this study, the level of knowledge and attitude towards family planning was relatively low and the level of family planning utilization was quite low in comparison with many studies. Every health worker should teach the community on family planning holistically to increase the awareness so that family planning utilization will be enhanced. Besides, more studies are needed in a thorough investigation of the different reasons affecting the non-utilizing of family planning and how these can be addressed are necessary.

## Introduction

Family planning (FP) is defined as a way of thinking and living that is adopted voluntary upon the bases of knowledge, attitude, and responsible decisions by individuals and couples [1]. Family planning refers to a conscious effort by a couple to limit or space the number of children they have through the use of contraceptive methods [2].

Family planning deals with reproductive health of the mother, having adequate birth spacing, avoiding undesired pregnancies and abortions, preventing sexually transmitted diseases and improving the quality of life of mother, fetus and family as a whole [3, 4].

The Federal Ministry of Health (FMOH) has undertaken many initiatives to reduce maternal mortality. Among these initiatives, the most important is the provision of family planning at all levels of the healthcare system [5, 6]. Currently, short-term modern family planning methods are available at all levels of governmental and private health facilities, while long-term method is being provided in health centers, hospitals and private clinics [6].

The study done in Jimma Zone, Ethiopia showed that good knowledge on contraceptives did not match with the high contraceptive practice [7]. Different researchers showed that the highest awareness but low utilization of contraceptives making the situation a serious challenge [8, 9].

Most of reproductive age women know little or incorrect information about family planning methods. Even when they know some names of contraceptives, they don’t know where to get them or how to use it. These women have negative attitude about family planning, while some have heard false and misleading information [10, 11] and the current study aimed in assessing the knowledge, attitude and practice (KAP) of FP among women of reproductive age group in South Achefer District, Northwest Ethiopia.

## Main text

### Methods and materials

#### Study design and setup

A community-based cross-sectional study was conducted in South Achefer District, Amhara Region, Northwest Ethiopia from March 01–April 01, 2017. Systematic sampling technique was used to recruit the sampled reproductive age women (15–49 years old). Based on the number of households obtained from the Kebele’s (Smallest administrative division) health post, the sample size (389) was distributed to the households. The sampling interval was determined based on the total number of 4431 households in the kebele. The first household was taken by lottery method and if there were more than one eligible individual in the same household one was selected by lottery method.

The data collection questionnaire was developed after reviewing different relevant literatures. The questionnaire, first developed in English language and then translated to Amharic (local language). Pretest was done on 5% of the total sample size at Ashuda kebele. After the pretest, necessary modifications and correction took place to ensure validity.

Those reproductive age women who answered ≥ 77% from knowledge assessing questions were considered as having good knowledge, those women who scored ≥ 90% from attitude assessing questions were considered as having favorable attitude and those women who scored ≥ 64% from practice assessing questions were considered as having good over all practice towards FP [7].

#### Data processing and analysis

The collected data was cleaned, entered and analyzed using SPSS version 21 software. Descriptive statistics were employed to describe socio-demographic, knowledge, attitude and practice variables. Chi squared (χ^2^) test was used to determine association between variables. Associations were considered statistically significant when P-value was, < 0.05.

### Results

#### Socio-demographic characteristics of participants

The response rate in this study was 97.9%. Among 381 participants included, 185 (49%) were from rural villages. About 47% of the participants were illiterate and 52% were completed primary education. The monthly household income of the majority (42.5%) of the participants was between 1000 and 3000 Ethiopian birr. Regarding the family size of the participant’s, majority (48.3%) of them had ≥ 3 children.

The mean age of participants was 29.7 ± 6.4. Two hundred forty six (64.6%) and 133 (34.9%) were house wife’s and farmers respectively by their occupation. Almost two-third (65.4%) of participants were married, 24.9% were divorced by their marital status (Table 1).

**Table 1:** Socio-demographic characteristics of study participants, South Achefer District, North West Ethiopia, 2017 (n = 381)

Variables	Frequency	Percent
Residence		
Rural	185	48.6
Urban	196	51.4
Age (years)		
< 18	38	10.0
18–29	139	36.5
≥ 30	204	53.5
Educational level		
Illiterate	179	47.0
Primary education	199	52.2
Secondary education	3	0.8
Monthly HH income		
< 1000 ETB	168	44.1
1000–3000 ETB	162	42.5
> 3000 ETB	51	13.4
Family size/no of children		
Have no children	45	11.8
Have 1 child	46	12.1
have 2 children	106	27.8
Have 3 or more children	184	48.3
Occupation		
House wife	246	64.6
Farmer	133	34.9
Daily laborer	2	0.5
Religion		
Muslim	3	0.8
Orthodox	378	99.2
Marital status		
Divorced	91	24
Married	249	65.4
Single	37	9.7
Widowed	4	0.9

The current currency exchange rate ($ 1 = 27.50 ETB)

*ETB* Ethiopian birr

#### Knowledge status of participants

All of participants ever heard about family planning methods. The major sources of information were from health workers (57.5%) and radio (41.5%). Regarding perceived side effects of using family planning, 13.1%, 24.9%, 9.7% and 52.2% of participants were responded heavy bleeding, irregular bleeding, an absence of menstrual cycle and abdominal cramp respectively were mentioned as a side effect. Among those who have children; 24.6% gave their last birth at home and 75.5% gave their last birth at the health institution. Regarding the overall knowledge of study participants, 161 (42.3%) had good knowledge towards family planning and the rest 220 (57.7%) had poor knowledge.

#### Attitude status of participants

The majority (88.5%) of the respondents ever discussed on family planning issues with their partners and wants to use it in the future. About 24.5% of the participants reported that they believe family planning exposes to infertility. Almost 23 (22.8%) of study participants reported that using family planning contradicts with their religion and culture. Regarding the overall attitude, 224 (58.8%) of the participants had favorable attitude and 157 (41.2%) had unfavorable attitude towards family planning.

#### Practice on family planning

Three fourth (75.3%) of study participants ever used contraceptive methods. The main types were pills (7.4%) and injectable (77.2%). The most common current reasons for not using were a desire to have a child (53.2%) and preferred method not available (46.8%). Almost half (50.4%) of study participants had good practice and the rest 49.6% had poor practice.

#### Factors associated with family planning practice

Study participants’ religion was not included in the analysis due to lack of variance, since almost all (99.2%) of participants were Orthodox Christians by their religion.

Women who had good knowledge were more likely to practice FP than those who have low knowledge (χ^2^ = 117.995, d.f. = 1, *P *< 0.001) and women who had favorable attitude towards FP were more likely to practice FP (χ^2^ = 106.696, d.f. = 1, *P *< 0.001). It was also seen that residence, age, educational status, occupation, marital status, number of children and monthly income of the were significantly associated with the practice of FP [(χ^2^ = 69.723, d.f. = 1, *P *< 0.001), (χ^2^ = 104.252, d.f. = 2, *P *< 0.002), (χ^2^ = 119.264, d.f. = 1, *P *< 0.001), (χ^2^ = 41.519, d.f. = 1, *P *< 0.001), (χ^2^ = 39.050, d.f. = 1, *P *< 0.001), (χ^2^ = 144,400, d.f = 3, *P *< 0.001) and (χ^2^ = 179.366, d.f. = 1, *P *< 0.002)] respectively (Table 2).

**Table 2:** Chi Square analysis result on FP practice and selected characteristics of participants, South Achefer district, 2017 (n = 381)

Explanatory variable	Had poor FP practice Number (%)	Had good FP practice Number (%)	Chai-square (X^2^)	*P* value
Residence				
Urban	56 (28.6%)	140 (71.4%)	69.723	< 0.001*
Rural	133 (71.9%)	52 (28.1%)		
Total	189 (49.6%)	192 (50.4%)		
Marital status				
Married	94 (37.8%)	155 (62.2%)	39.050	< 0.001*
Single and widowed	95 (72.0%)	37 (28.0%)		
Total	189 (49.6%)	192 (50.4%)		
Educational level				
Illiterate	142 (79.3%)	37 (20.6%)	119.264	< 0.001*
Primary and secondary	47 (23.3%)	155 (76.7%)		
Total	189 (49.6%)	192 (50.4%)		
Age category (years)				
< 18	36 (94.7%)	2 (5.3%)	104.252	< 0.001*
18–29	100 (71.9%)	39 (28.1%)		
≥ 30	53 (26.0%)	151 (74.0%)		
Total	189 (49.6%)	192 (50.4%)		
Occupation				
House wife	91 (36.9%)	155 (63.1%)	41.519	< 0.001*
Farmer	98 (72.6%)	37 (27.4%)		
Total	189 (49.6%)	192 (50.4%)		
Knowledge				
Less knowledgeable	162 (73.6%)	58 (26.4%)	117.995	< 0.001*
Knowledgeable	27 (16.8%)	134 (83.2%)		
Total	189 (49.6%)	192 (50.4%)		
Attitude				
Unfavorable attitude	128 (81.5%)	29 (18.5%)	106.696	< 0.001*
Favorable attitude	61 (27.2%)	163 (72.8%)		
Total	189 (49.6%)	192 (50.4%)		
Family size (no of children)				
Have 1 child	32 (71.1%)	13 (28.9%)	144.400	< 0.001*
Have 2 children	11 (23.9%)	35 (76.1%)		
Have 3 children	99 (93.4%)	7 (6.6%)		
Have 4 or more children	47 (25.5%)	137 (74.5%)		
Total	189 (49.6%)	192 (50.4%)		
Monthly HH income				
< 1000 ETB	129 (76.8%)	39 (23.2%)	179.366	< 0.001*
1000–3000 ETB	16 (9.9%)	146 (90.1%)		
> 3000 ETB	44 (86.3%)	7 (13.7%)		
Total	189 (49.6%)	192 (50.4%)		

* Significant P-value

### Discussion

Increasing program coverage and access of family planning will not be enough unless all eligible women have adequate awareness for favorable attitude and correctly and consistently practicing as per their need. Increasing awareness/knowledge and favorable attitude for practicing FP activities at all levels of eligible women are strongly recommended [6].

The results of the present study showed that 42.3% of study participants had good knowledge, 58.8% had favorable attitude, and 50.4% had good practice towards family planning. This finding was lower than a study conducted in Jimma zone, Southwest Ethiopia [7], Sudan [9], Tanzania [12] and another study done in Rohtak district, India [13]. The difference may be due to; studies done in Jimma zone, Sudan, Tanzania and Rohtak district involve only those coupled/married women. Married women might have good knowledge and attitude for practicing family planning. But in the current study, all women of reproductive age group regardless of their marital status were studied and this may lower their knowledge and attitude.

The current study showed that, 50.4% of reproductive age women were practicing family planning which was almost in line with a study done in Cambodia [14] and higher than a study done in rural part of Jordan [15] and India [16]. But it was lower than studies conducted in Jimma zone, Ethiopia [7], Rohtak district, India [13], urban slum community of Mumbai [17] and in Sikkim [18] in which 64%, 62%, 65.6% and 62% of participants respectively used family planning. The difference might be due to that study participants in Jimma zone, Rohtak and Mumbi were relatively residing in large city/town and this may help them to have a better access for family planning compared to the study done in South Achefer District.

In the current study, urban residents were more likely to use family planning methods (71.4%) than their rural counterparts (28.1%). This finding was in line with the findings from Ethiopian Demographic Health Survey (EDHS) [2]. This might be due to the reason that urban residents are more aware of family planning and hence practicing better.

It has also found that women who completed primary & secondary education were practicing family planning than those who were uneducated (77.1% and 20.6%) respectively. This finding was in line with a study done in Jimma, Ethiopia [19]. This might be due to the fact that women who were able to read and write would think in which FP activities are useful to be economically, self-sufficient and more likely to acquire greater confidence and personal control in marital relationships including the discussion of family size and contraceptive use.

This study showed that, age of the study participants had an association with practicing FP. Those reproductive age women’s whose age > 30 years were practicing family planning better than those whose age < 18 years. This finding was in line with a study done in India [20]. This might be due to the reason that, when age increases mothers awareness, attitude and practice towards family planning may increase. In addition, as age increases the chance of practicing sexual intercourse increases and as a result they would be interested to utilize family planning in one or another way.

It has also revealed that women’s average monthly household income has an association with their FP practicing habit. Those study participants whose average monthly income < 1000 ETB were using FP better than whose average monthly income > 3000 ETB. This is might be because those relatively who had better income may need more children and those with low income may not want to have more children beyond their income.

The current study also showed that knowledge and attitude of reproductive age women were related to FP utilization. Those reproductive age women who had good knowledge were utilized FP better than from those who were less knowledgeable. Those participants with favorable attitude were practicing better than those who had unfavorable attitude. This is might be due to the fact that knowledge and attitude for specific activities are the key factors to start behaving and maintaining it continuously.

### Conclusion and recommendation

The level of knowledge and attitude towards family planning was relatively low and the level of family planning utilization was quite low in comparison with many studies.

Study participant’s residence, marital status, educational level, occupation, age, knowledge, attitude, their family size and their monthly average income were associated with FP utilization habit of reproductive age women.

Every health worker should teach the community on family planning holistically to increase the awareness so that family planning utilization will be enhanced.

Besides, more studies are needed in a thorough investigation of the different reasons affecting the non-utilizing of family planning and how these can be addressed are necessary.

## Limitation of the study

As the data were collected using interviewer administered questionnaire, mothers might not felt free and the reported KAP might be overestimated or underestimated.

We do not used qualitative method of data collection to gather study participant’s internal feeling about family planning, so that triangulation was possible. In addition, barriers for utilizing contraception not addressed.

## Abbreviations

EDHS: Ethiopian Demographic Health Survey
ETB: Ethiopian birr
FMOH: Federal Ministry of Health
FP: family planning
KAP: knowledge, attitude and practice
